# Analysis of gene expression changes upon topobexin treatment and TOP2B-knockout in hiPSC-derived cardiomyocytes

**DOI:** 10.1242/bio.062592

**Published:** 2026-08-12

**Authors:** Veronika Kerestes, Ian Cowell, Anna Jirkovska, Mushtaq Khazeem, Galina Karabanovich, Iuliia Melnikova, John Casement, Jan Kubes, Tomas Simunek, Jaroslav Roh, Matthew Schellenberg, Adam Creigh, Chunbo Yang, Majlinda Lako, Lyle Armstrong, Caroline Austin

**Affiliations:** ^1^ Charles University, Faculty of Pharmacy, Hradec Kralove, 500 03, Czech Republic; ^2^ Newcastle University, Faculty of Medical Sciences, Newcastle upon Tyne, NE1 7RU, UK; ^3^ Department of Molecular Biology, Mustansiriyah University, Baghdad 10015, Iraq; ^4^ Department of Biochemistry and Molecular Biology, Mayo Clinic, Rochester, MN 55905, USA

**Keywords:** TOP2, TOP2B, Gene expression, Cardiomyocyte, hiPSC

## Abstract

The role of DNA topoisomerase II beta (TOP2B) in cardiomyocyte differentiation is poorly understood. To address this, human induced pluripotent stem cells (hiPSC) were differentiated into cardiomyocytes (CM) that were wild type (WT) or contained a genomic deletion of Topoisomerase 2B (BKO). Both WT and BKO hiPSC could be induced to differentiate into sheets of beating cardiomyocytes. BKO hiPSC take slightly longer to differentiate into sheets of beating CM than WT iPSC. RNA was prepared from both undifferentiated and differentiated WT and BKO hiPSC. RNA-seq was used to examine gene expression changes when the WT and BKO hiPSC were differentiated into CM. Gene expression changes following differentiation of BKO cells were largely similar to those in WT cells. In addition, the differentiated WT CM were treated with dexrazoxane (ICRF-187), a TOP2 catalytic inhibitor that targets both TOP2A and TOP2B, or topobexin, a new TOP2B selective catalytic inhibitor. Topobexin inhibition partially phenocopied a TOP2B deletion and thereby providing an alternative to TOP2B gene knockout in many cell lines. In future, hiPSC derived CM with and without TOP2B and inhibition by topobexin *ex vivo* CM could be used to study anthracycline-induced cardiotoxicity and to screen for cardioprotectants.

## INTRODUCTION

DNA topoisomerase II beta (TOP2B) is an essential nuclear enzyme that modulates DNA topology by introducing transient double-strand breaks. Unlike the alpha isoform (TOP2A), which is primarily active in proliferating cells, TOP2B is expressed ubiquitously, including in post-mitotic cells, such as neurons and cardiomyocytes. TOP2B facilitates the resolution of topological constraints during transcription and participates in long-range chromatin remodelling and gene activation (Pommier et al., 2022; Cowell et al., 2023). TOP2B is involved in regulating transcription (Austin et al., 2021) but is not essential for cell viability (Dereuddre et al., 1995; Errington et al., 1999; Toyoda et al., 2008). However, TOP2B is required in some differentiation pathways including neuronal and B cell differentiation (Tiwari et al., 2012; Madabhushi, 2018; Khazeem et al., 2022; Broderick et al., 2019).

Using TOP2B^−/−^ embryonic fibroblast cell lines (Lyu et al., 2007) showed a connection between TOP2B and the prevention of anthracycline cardiotoxicity with dexrazoxane (ICRF-187). A link between TOP2B and anthracycline induced cardiotoxicity was confirmed by (Zhang et al., 2012) using an *in vivo* conditional murine cardiomyocyte-specific knockout study that firmly established TOP2B as the causal driver of anthracycline-induced cardiotoxicity (Fabiani et al., 2025). Two studies have used iPSC derived cardiomyocytes to investigate the role of TOP2B on anthracycline-induced cardiotoxicity: one study used CRISPR-Cas9 deletion of TOP2B (Maillet et al., 2016) and a second used siRNA knockdown of TOP2B (Saroj et al., 2025). These studies showed that deletion or knockdown of TOP2B reduced the loss of viability of the cells following exposure to doxorubicin. Furthermore, Saroj et al. showed that siRNA knockdown was more effective than (ICRF-187) treatment, and they suggested siRNA mediated TOP2B inhibition could provide a way to mitigate doxorubicin cardiotoxicity. However, methods for effective delivery of siRNA to cardiomyocytes *in vivo* have not yet been established, and a small molecule drug would be more clinically achievable approach. Notably, topobexin a specific TOP2B inhibitor has recently been reported (Kubeš et al., 2025).

In this study, we developed human induced pluripotent stem cells (hiPSC) with CRISPR-Cas9 deletion of TOP2B (BKO) and differentiated them into beating cardiomyocytes. In the selected clone (line 18D) we performed transcriptomic profiling to evaluate gene expression changes before and after differentiation. In addition, we compared the transcriptional effects of TOP2B genetic deletion with the effects of its pharmacological inhibition by the non-isoform-selective inhibitor ICRF-187 and by a novel TOP2B selective catalytic inhibitor topobexin (Kubeš et al., 2025). By comparing genetic inactivation with pharmacological inhibition, we aimed to define the role(s) of TOP2B in cardiomyocyte differentiation and transcriptional regulation for exploring how modulation of TOP2B activity may influence cardiotoxic responses and broader aspects of cardiac biology.

## RESULTS

### Generation of knockout hiPSC

TOP2B null hiPSC clones were generated from wild-type (WT) hiPSC cells previously reported (AD1/WT1) (Tsikandelova et al., 2024) using CRISPR-Cas9 as previously described for SH-SY5Y neuroblastoma cells using TOP2B exon 1 guide RNA 6 (Khazeem et al., 2022) (Fig. S1A). TOP2B knockout was confirmed by DNA sequencing and by western blotting the selected clones. Data for clone 18D are shown in Fig. S1B and C. TOP2B null lines (BKO) appeared phenotypically indistinguishable to the WT with identical appearance and growth properties. However, differential gene expression comparisons between the undifferentiated TOP2B null cell line and the WT cell line did reveal some transcriptional changes. These are highlighted in a volcano plot in Fig. S1D. With a cut-off of a twofold change in transcript abundance, 101 genes were significantly downregulated genes and 426 upregulated in the TOP2B null hiPSC line.

### Differentiation of WT and knockout iPSC

**Fig. 1.**
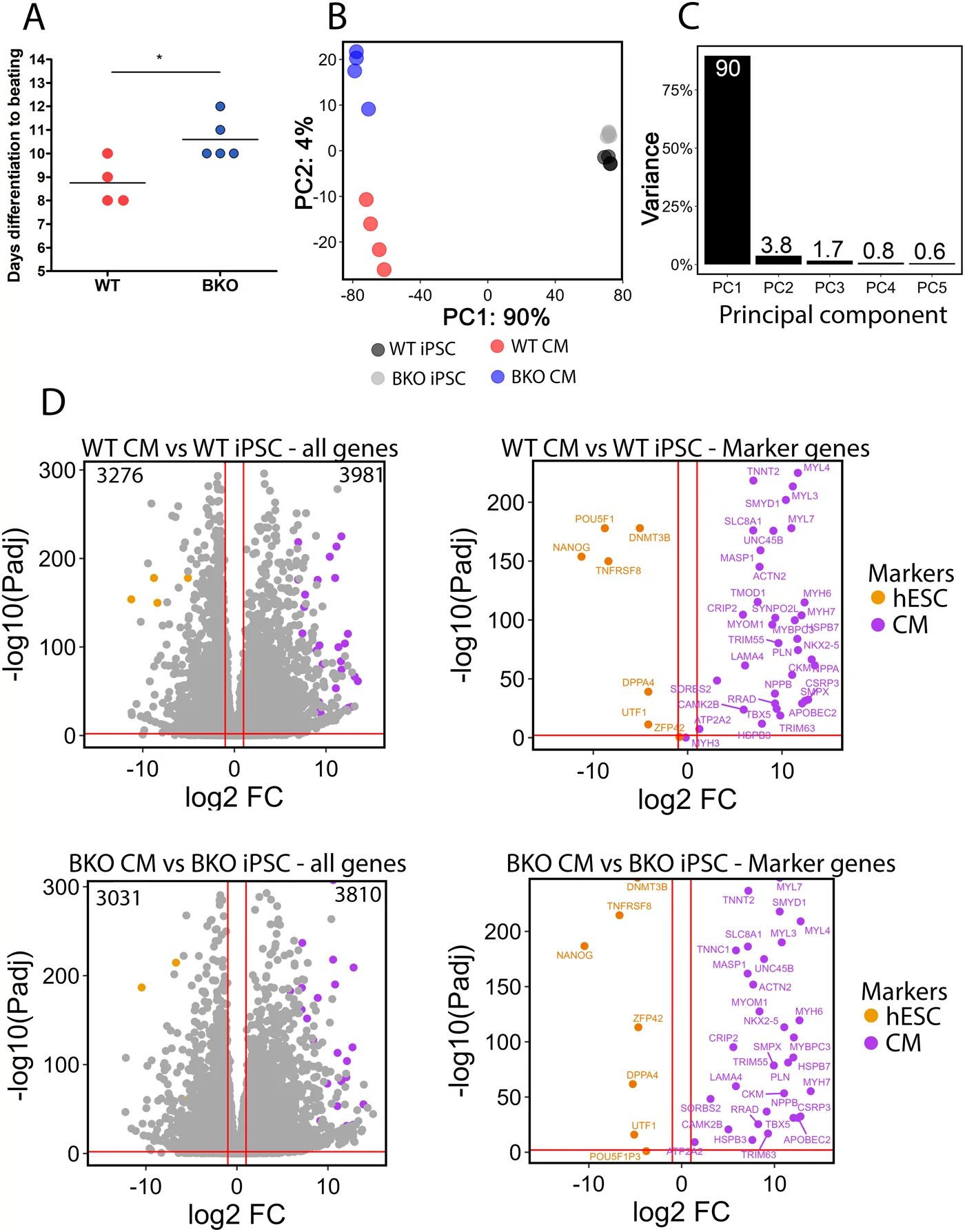
**WT and TOP2B null hiPSCs can efficiently differentiate into beating cardiomyocytes.** (A) The differentiation protocol resulted in the conversion of monolayers of WT or TOP2B null (line 18D, BKO) hiPSC cells into layers of beating cardiomyocytes in 8-12 days. However, on average this took 1 day longer for BKO than WT hiPSC cells. Each spot represents a separate hiPSC to CM differentiation experiment, four biological replicates for WT and five biological replicates for the BKO. Line=mean. Statistical significance was determined using a two-tailed unpaired *t*-test, *P<0.05. (B) Principal component analysis of RNA-seq analysis comparing WT and TOP2B null (BKO) hiPSC and derived CM (quadruplicate biological replicates). (C) Scree plot showing the variance of PC1-PC5 (D) Volcano plots to visualize gene expression changes upon differentiation of WT and BKO hiPSC cells to CM. Red lines represent cutoffs of >2-fold change in transcript abundance with a *P*adj of <0.05. Numbers in the corners of the plots highlight the number of up- or downregulated protein-coding genes in the CM compared to the hiPSC cells, using the above cutoffs.

hiPSC were differentiated using a protocol derived from that reported by Lian et al. (2013). Both WT and BKO hiPSC differentiated efficiently and reproducibly using this protocol. Four biological replicates for the WT hiPSCs and five biological replicates for the BKO hiPSCs. However, differentiation to the endpoint of beating sheets of cardiomyocytes (CM) took approximately 1 day longer for the TOP2B null cells after the replacement of mTeSR1 with RPMI 1640+B27+GSK3 inhibitor (CHIR 99021)+Activin A (see Fig. 1A). The time until beating for each cell line and each replicate is shown in Fig. 1A. The mean time to beating for WT CM was 9 days and for BKO CM it was 10.5 days. Videos of beating sheets of differentiated WT and BKO 18D are shown as Movie 1 and 2 (BKO18D and WT).

**Table 1.** hiPSC differentiation protocol

Day	Medium	Growth factors
1	RPMI 1640+B27 without insulin	10 μM CHIR 99021, 10 ng/ml Activin A
2	RPMI 1640+B27 without insulin	None
4	RPMI 1640+B27 without insulin	5 μM IWP2
6	RPMI 1640+B27 without insulin	None
8	RPMI 1640+B27 with insulin	None
Every other day	RPMI 1640+B27 with insulin	None

### Differential expression analysis confirmed the differentiation of WT and BKO cell lines-increased expression of CM marker genes

A comparative transcriptome analysis was performed in WT hiPSC and hiPSC null for TOP2B (BKO hiPSC) that were generated by targeted gene knockout. RNA-sequencing was performed on four biological replicates for each condition [undifferentiated WT hiPSC, undifferentiated BKO hiPSC, WT differentiated into cardiomyocytes (WT CM), BKO differentiated into cardiomyocytes (BKO CM)]. Fig. 1B shows principal component analysis for the four conditions and the variance is shown. This shows the biggest difference is between the undifferentiated cell lines and the differentiated cell lines. The undifferentiated cell lines clustered together away from the differentiated cells. Fig. 1C shows a scree plot showing 90% of the variance is in PC1, 3.8% in PC2, 1.7% in PC3, 0.8 in PC4 and 0.6 in PC5. As expected, the largest difference in gene expression profiles was between the hiPSC and derived CM cells of both genotypes (PC1); but notably the four WT CM replicates cluster separately from the four BKO CM samples (PC2). This is consistent with a largely shared pattern of transcriptional changes upon differentiation to the CM state, but with some differences between the WT and BKO cells in this respect. The differential gene expression between the undifferentiated and differentiated cell lines is illustrated in the volcano plots in Fig. 1D. Comparison of the differentiated and undifferentiated cell lines showed that similar numbers of genes are up or down regulated upon differentiation in WT or BKO cell lines (red lines indicate twofold change in gene expression). Pluripotency (human embryonic stem cell, hESC) and cardiomyocyte marker genes have previously been identified in global expression profiles of highly enriched cardiomyocytes derived from human embryonic stem cells in Xu et al. (2009) (Table 2). As expected, cardiac marker genes were upregulated in CM cells of both genotypes. These genes included *ACTN2* (alpha actinin 2), *MYL7* (Myosin light chain 7) and *TNNT2* (troponin 2, cardiac type). Similarly, markers of pluripotency including *NANOG*, *DPPA4* and *DNMT3B* were downregulated in both WT and BKO CM cells Fig. 1D.

**Table 2.** **CM and pluripotency (hESC) marker genes, from** **Xu et al. (2009)**

Gene symbol	Type
ACTN2	CM
APOBEC2	CM
ATP2A2	CM
CAMK2B	CM
CKM	CM
CRIP2	CM
CSRP3	CM
HSPB3	CM
HSPB7	CM
LAMA4	CM
MASP1	CM
MB	CM
MYBPC3	CM
MYH6	CM
MYL3	CM
MYL4	CM
MYL7	CM
MYOM1	CM
PLN	CM
PPP1R12B	CM
PPP1R14C	CM
RRAD	CM
SLC8A1	CM
SMPX	CM
SMYD1	CM
SORBS2	CM
SRD5A2L2	CM
SYNPO2L	CM
TMOD1	CM
TNNC1	CM
TNNT2	CM
TRIM55	CM
TRIM63	CM
UNC45B	CM
NPPA	CM
NPPB	CM
TBX5	CM
MYH7	CM
NKX2-5	CM
TNNC1	CM
NANOG	hESC
ZFP42	hESC
POU5F1	hESC
TNFRSF8	hESC
DPPA4	hESC
UTF1	hESC
DNMT3B	hESC
LIN28	hESC

### Differences in gene expression between WT and BKO CM

**Fig. 2.**
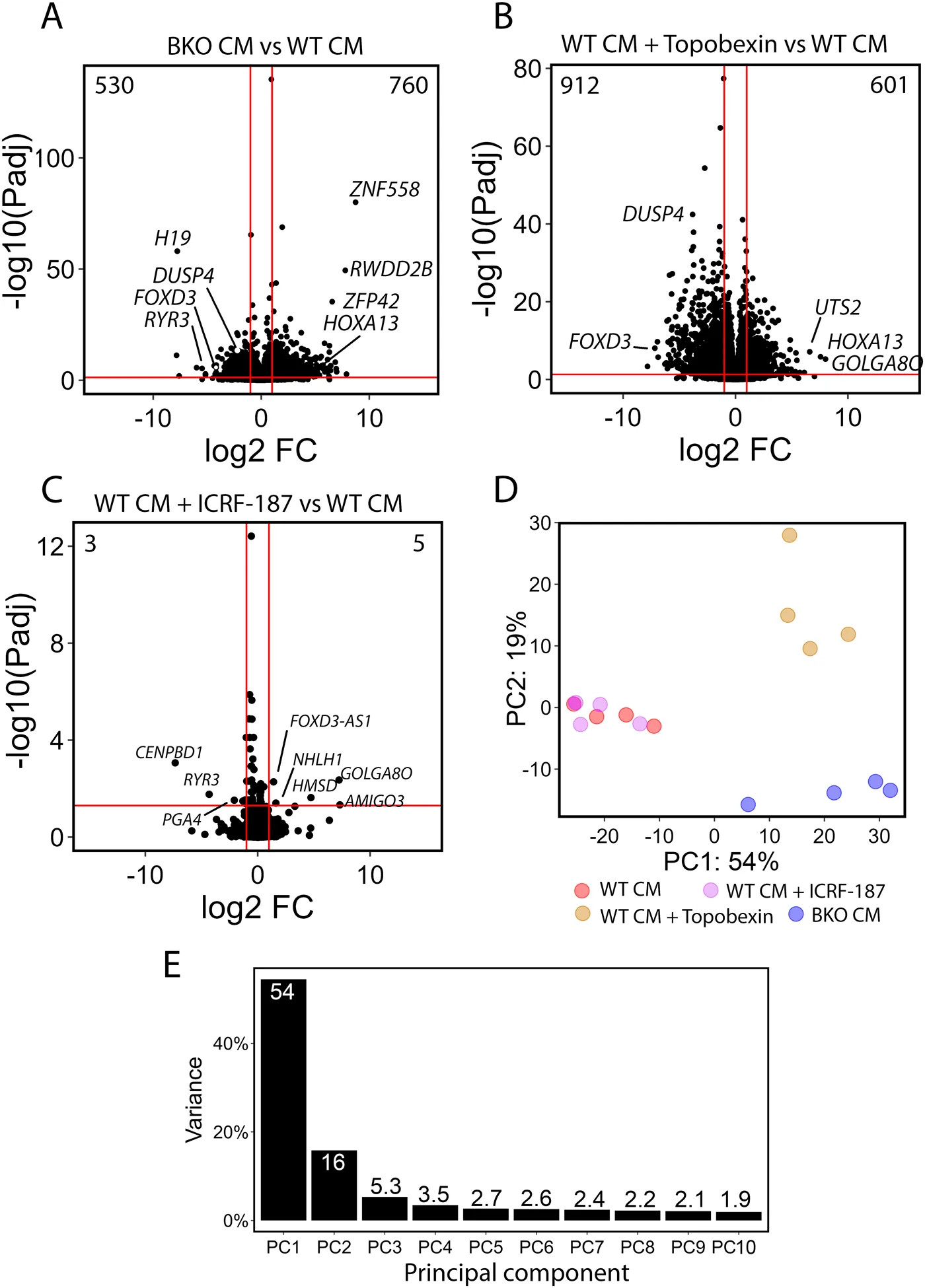
**Transcriptome comparison of WT and BKO hiPSC-derived CM cell, and effect of TOP2 inhibitors topobexin and ICRF-187.** (A) Volcano plot illustrating the number of genes up- or downregulated in BKO versus WT CM. (B) Effect of topobexin gene expression (transcript abundance) in WT CM. (C) Effect of ICRF-187 on gene expression (transcript abundance) in WT CM. For volcano plots, cut-offs (red-lines) were as in Fig. 1. Representative genes are highlighted. (D) Principal component analysis of transcript abundance comparing in four conditions, WT and TOP2B null (BKO) CM, WT CM treated with topobexin and CM treated with ICRF-187 (quadruplicate biological replicates). (E) A scree plot of PC1-PC10.

Although there were a similar number of gene expression changes when either WT or BKO hiPSC differentiated into beating CM (Fig. 1D), differential gene expression comparisons between the differentiated BKO CM and WT CM showed 530 genes were downregulated and 760 upregulated in the BKO CM compared to WT CM (Fig. 2A), so there are slightly more upregulated than downregulated genes. None of the CM markers were affected significantly comparing WT to BKO CM, nor were the hESC genes, except for *ZFP42*, which was more abundant in the BKO CM compared to WT. However, this gene is expressed at a much higher level in the BKO hiPSC than in their WT equivalents (see Table S1). Comparing genes whose expression changed during differentiation (≥2-fold change, *P*adj <0.05), the majority of changes (5531) were shared between both genotypes. However, 1672 and 1267 genes were differentially expressed only in WT or BKO, respectively, during differentiation to CM (see Fig. S2).

### Effect of topoisomerase II inhibition on gene expression in the WT CM

To compare topoisomerase II inhibition with TOP2B knockout WT CM were treated with two catalytic inhibitors: topobexin (Kubeš et al., 2025), a recently developed small molecule with selectivity for TOP2B (10 μM, 3 h, Fig. 2B) or dexrazoxane (ICRF-187), a topoisomerase II inhibitor used clinically as a cardioprotectant (100 μM, 3 h, Fig. 2C) using concentrations where we see robust TOP2B inhibition and efficacy in cardiomyocytes as previously reported in our topobexin paper Fig. 2B and C show volcano plots of the differential gene expression patterns following treatment with topobexin or ICRF-187. Topobexin had a large effect, with 912 downregulated and 601 upregulated genes. In contrast, ICRF-187 had very little effect on gene expression in WT CM. None of the CM or hESC marker genes were significantly affected, and even taking the whole transcriptome, only eight genes were significantly altered in ICRF-187 treated cells (see Fig. 2C). A table of relative gene expression values (TPM values) for each condition is given in (Table S1). Notably, all of the genes whose expression was significantly altered by ICRF-187 were expressed at relatively low levels (Table S1). Transcripts corresponding to one of the genes upregulated by ICRF-187 (*GOLGA8O*) were also increased in abundance in BKO CM and topobexin-treated WT CM (Fig. 2, Table S1). Genes with a large increase in transcript abundance in the BKO CM included *ZNF588*, *RWDD2B*, *HOXA13* and the previously mentioned *ZFP42* and *GOLGA8O*. Of these selected genes, *HOXA13* and *GOLGA8O* were also elevated in topobexin-treated cells, while *ZNF588*, *RWDD2B* and *ZFP42* were not. Conversely, transcript abundance for genes such as *UTS2* was increased only in the topobexin-treated CM. Looking at selected downregulated transcripts, one of the most under expressed transcripts in the BKO cells, *H19*, is not changed in abundance in topobexin treated CM, while *DUSP4* and *FOXD3* were downregulated in both BKO and topobexin-treated cells.

**Fig. 3.**
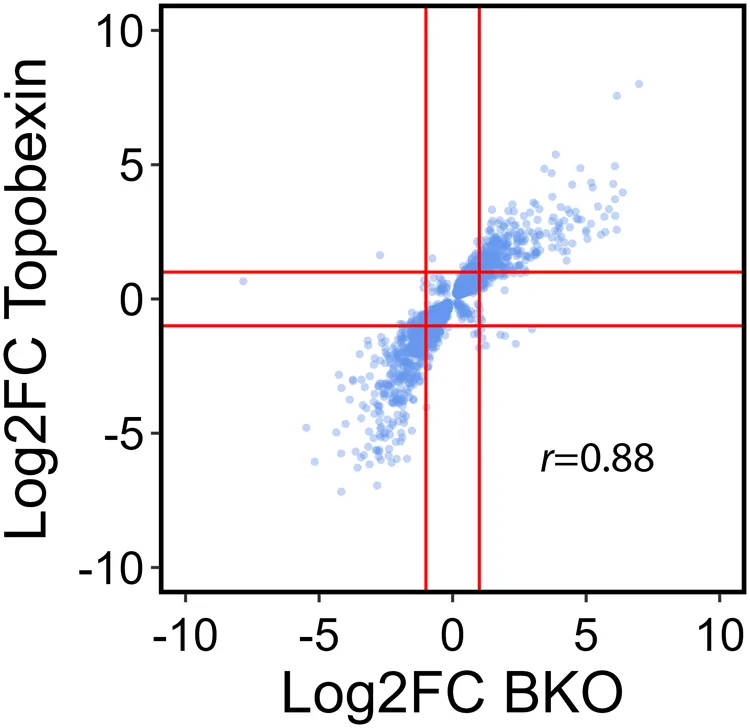
**Comparison of gene expression changes in WT versus BKO CM and topobexin treated versus untreated CM.** Differential gene expression tables were filtered to include only genes that change significantly (*P*adj <0.05) in expression in both TOP2B null CM versus WT cells and after treatment of WT CM with topobexin. The gene list was further filtered to only include protein-coding genes. Log2 FC values are plotted on the x and y axes. *r*=Pearson correlation coefficient.

A principal component analysis was carried out on the four differentiated conditions (untreated WT CM, BKO CM and WT CM cells treated with ICRF-187 or topobexin). For ICRF-187 treated WT CM, all four ICRF-187-treated CM replicates clustered together with the untreated WT CM, consistent with the limited transcriptional changes highlighted in Fig. 2C. In contrast, topobexin treated WT CM and BKO CM were separated from the untreated/ICRF-187 cluster by PC1 (54%), although they were separated from each other along the PC2 axis (Fig. 2D). PC1-PC10 is shown in Fig. 2E. About half of the genes whose expression was changed in the TOP2B null CM compared to WT, were also changed in the topobexin treated WT CM – consistent with the PCA analysis (Fig. S2B). However, fewer than half of the topobexin affected genes were also altered in the knockout (Fig. S2B). Of the genes whose expression changed in both conditions (WT versus BKO CM cells and WT CM cells versus WT CM cells+topobexin) we observed a significant correlation in the size and direction of the change in transcript abundance (Pearson correlation coefficient=0.88, Fig. 3). This suggests that topobexin partially phenocopies the BKO cells.

### RNA expression levels in the six conditions tested

**Fig. 4.**
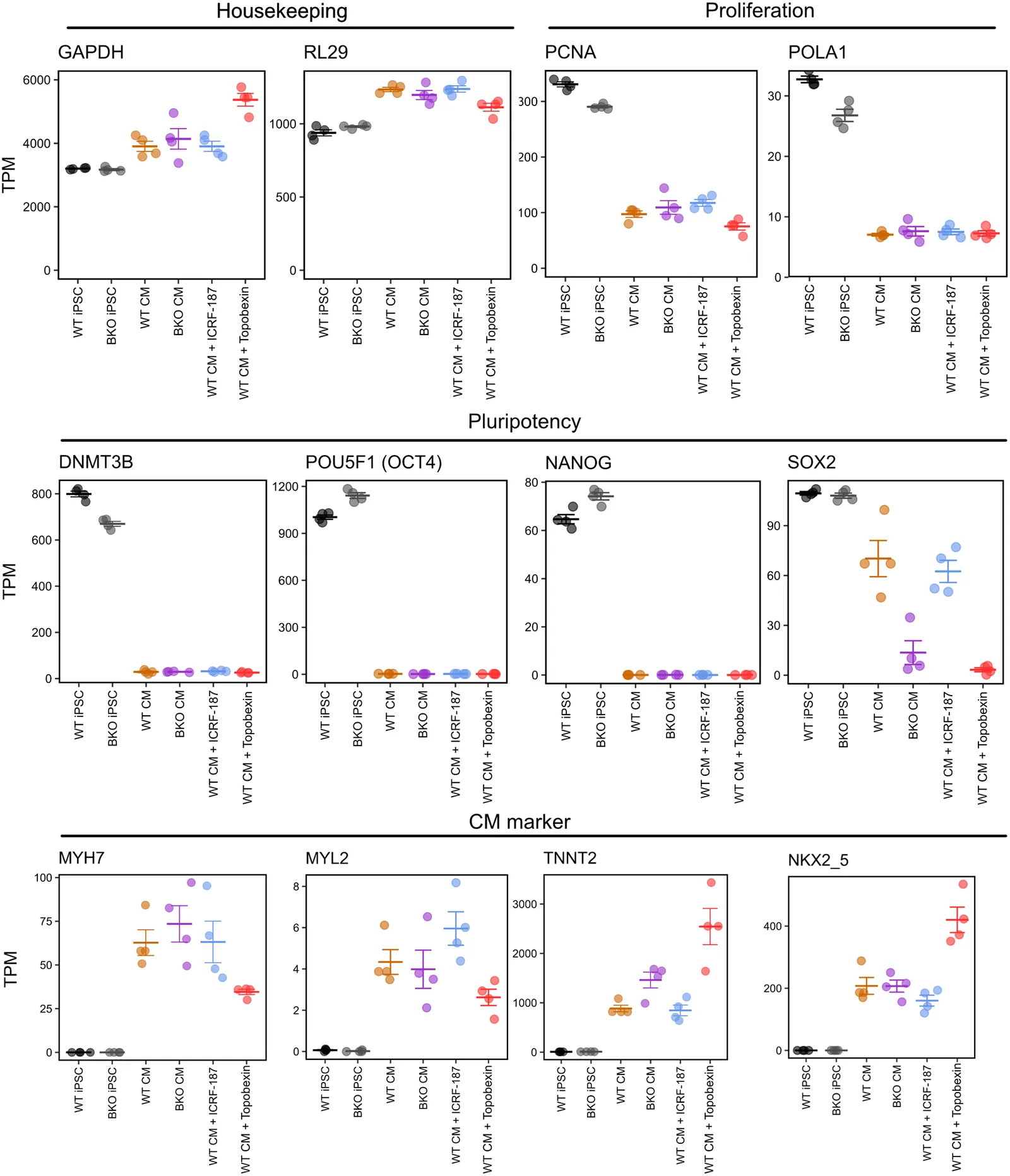
TPM levels in the RNA seq datasets for each of the six conditions analyzed.

Fig. 4 shows the TPM values for a selection of marker genes in all six conditions analysed. Two housekeeping genes were analysed GAPDH and RL29. Two markers of proliferation PCNA and POLA1, these showed the highest TPM values in the WT undifferentiated cell line and the BKO undifferentiated cell line. Four pluripotency marker genes are shown, all four show the highest TPM values in the undifferentiated cell lines. Three DNMT3B, POU5F1 (OCT4) and NANOG all reduced to very low levels in the CMs and this was not altered by the action of TOP2 inhibitors. SOX2 shows a different pattern with very low TPM levels in the BKO CM and WT CM treated with topobexin, but intermediate levels in the WT CM and WT CM treated with ICRF187. Four cardiac marker genes MYH7, MYL2, TNNT2 and NKX2-5 are also shown in Fig. 4, as expected these are all very low or absent in the undifferentiated WT and BKO, expression of all four increased in the differentiated CMs.

### Gene Set Enrichment Analysis

**Fig. 5.**
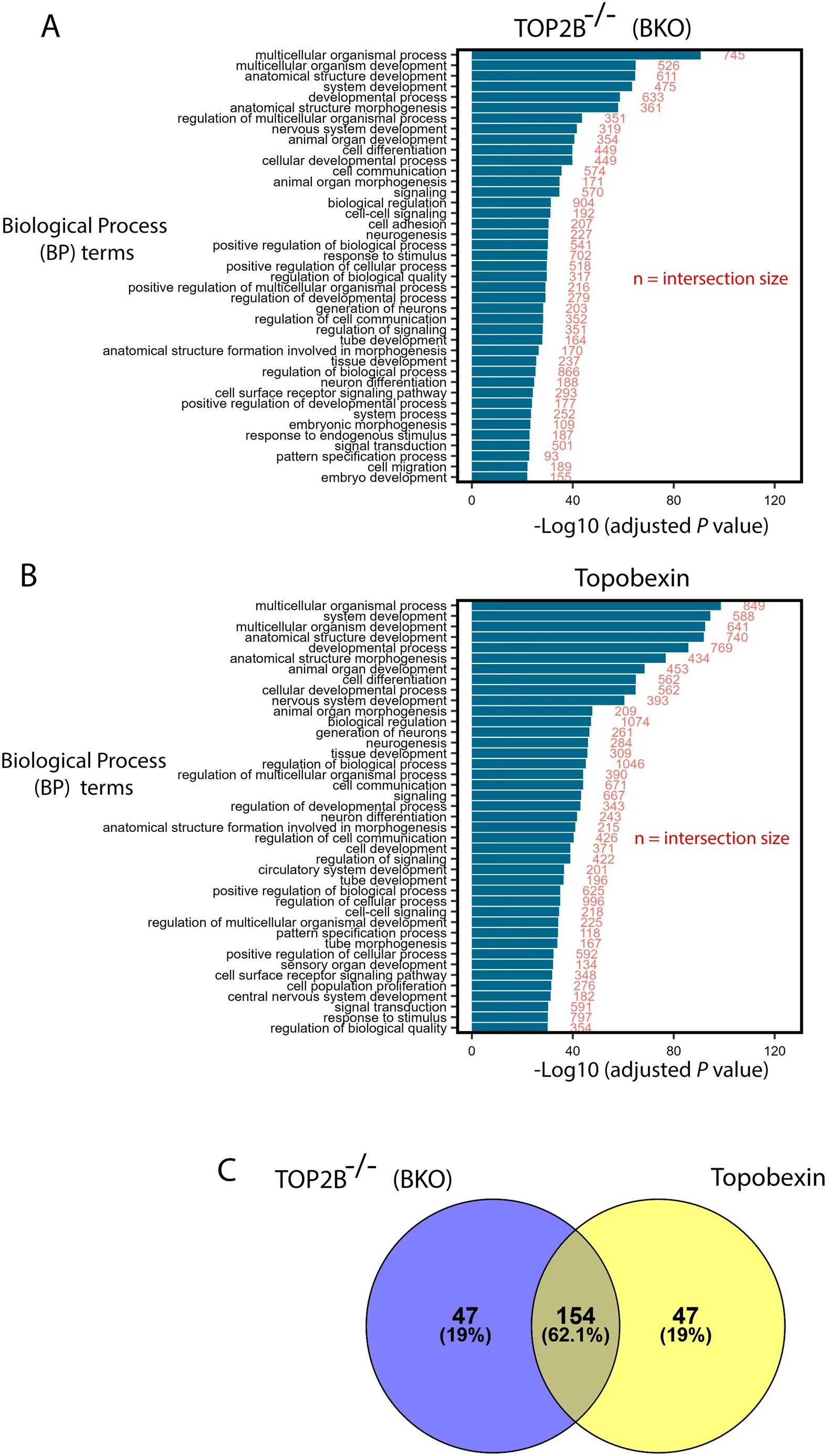
**Gene set enrichment analysis.** Sets of transcripts whose abundance changed (≥2 fold) in either TOP2B^−/−^ (BKO) CM or topobexin-treated CM compared to untreated WT CM were examined by g:GOSt functional enrichment analysis (https://biit.cs.ut.ee/gprofiler/gost). The top 41 biological pathway (BP) terms for (A) TOP2B^−/−^ (BKO) CM and (B) topobexin-treated CM were plotted by negative log_10_(adjusted *P*-value). Numbers in red correspond to the intersection size. (C) Venn diagram illustrating the degree of overlap between the top 200 (by statistical significance) BP terms highlighted for each condition.

We carried out Gene Set Enrichment Analysis using g:GOSt (Kolberg et al., 2023) in order to determine whether any specific biological pathways or process where disproportionately represented in the sets of genes whose transcript abundance significantly changed in either BKO CM or topobexin-treated CM compared to untreated WT CM. A large number of biological processes (BP) terms were highly significantly enriched for both gene sets. The BP terms most enriched related to cellular and tissue development and differentiation. The top 40 scoring BP terms are plotted by significance value in Fig. 5 and are very similar for BKO and topobexin inhibition. The entire data set is present in Table S2.

### Effect of removal of TOP2B on gene expression of long genes in the CM

TOP2B has been reported to be involved in the transcription of long genes (Austin et al., 2021). For example, in SH-SY5Y cells, the longest genes were more likely to be downregulated in TOP2B null compared to the WT cells (Khazeem et al., 2022). To determine if this also occurs in CM an equivalent analysis was carried out for the WT versus BKO CM and for control versus topobexin-treated cells. As we observed previously, downregulated transcripts were slightly more common in the upper quartile of gene lengths (Table 3), although this effect was not as pronounced as observed when comparing WT to TOP2B null SH-SY5Y cells. In WT versus BKO CM, downregulated genes were least abundant in the middle two quartiles, and this pattern was also present in topobexin-treated cells. Taken together the data in Figs 2, 3 and Table 3 support the idea that topobexin partially phenocopies TOP2B gene inactivation in hiPSC-derived CM.

**Table 3.** Relationship between gene length and probability of a gene being down regulated in TOP2B null or topobexin-treated CM (cut-offs, *P*adj <0.05, Log2FC<−1)

	WT versus BKO (TOP2B null)			WT versus WT+Topobexin	
Gene length quartile	Number of genes	Percentage of downregulated genes		Number of genes	Percentage of downregulated genes
Q1	152	28.7		239	26.5
Q2	108	20.4		198	22.0
Q3	96	18.1		184	20.4
Q4	174	32.8		280	31.1
Total	530	100		901	100

## DISCUSSION

Classical *in vitro* cardiac models often rely on cell lines or primary CM derived from non-human species such as rodents. *In vitro* models of CM are indispensable tools for studying cardiac physiology, molecular signaling, and responses to external stimuli. These models support both basic research and applied biomedical studies, including drug development and safety testing. However, while these systems are accessible and well-established, they may not fully replicate human-specific molecular mechanisms, electrophysiological properties, or gene regulation patterns. Cross-species differences can limit the translational value of findings, particularly when investigating subtle regulatory processes or drug responses that are specific to human biology. Immortalized CM-like cell lines and rodent primary CM present additional limitations. For example, cell lines frequently exhibit altered signaling pathways and an immature cardiac phenotype. Primary CM, although more physiologically relevant, are difficult to isolate in large numbers, are short-lived in culture, and generally cannot be propagated. These challenges underscore the need for more physiologically relevant and genetically tractable human cell-based models. Many diseases or pathophysiological mechanisms seen in human medicine are difficult to model in experimental cellular or animal systems reproducibly and directly translatable into clinical situation (Mandenius et al., 2011).

Human-derived hiPSCs have numerous advantages over animal models or immortalized cell lines. They can be differentiated into a variety of cells including CM (Bourque et al., 2022). Also, they can be used in high-throughput screening (Zink et al., 2020), patient-specific research or personalized medicine (Pang, 2020), and the response to drugs can be explored in cardiac organoids (Prondzynski et al., 2024). Thus, hiPSC offer a compelling alternative. Derived from adult somatic cells, hiPSC-CM provide a renewable, human-specific model system that allows for genetic manipulation and disease modelling in a controlled setting. They can reproduce many key structural and functional features and may be used to investigate developmental processes, gene regulation, and cellular responses in a human context.

TOP2B has also been implicated in the mechanism of anthracycline-induced cardiotoxicity, although generally the role of TOP2B in human CM remains insufficiently characterized. The use of hiPSC-CM provides a valuable opportunity to study the function of TOP2B in a controlled human cellular context. We have shown that iPSC deleted for TOP2B can be differentiated into CM, as previously reported (Maillet et al., 2016). We have extended this by carrying out a transcriptomics analysis to determine the differential gene expression in the undifferentiated and differentiated cells, both with and without TOP2B and this data has been deposited in gene expression omnibus to enable other researchers to access to this data. Four biological replicates were sequenced where RNA was extracted from independently differentiated wells of BKO iPSC clone 18D. However, the four replicates were derived from the same clonal line, which represents a potential limitation of this study. Although the BKO iPSC could differentiate efficiently and reproducibly into beating CM this took slightly longer than the WT iPSC suggesting some differences in the differentiation of WT and BKO into CM. Consistent with the observed CM phenotype (sheets of beating cells), both WT CM and BKO CM exhibited induced CM marker genes and silencing of pluripotency marker genes. However, there were significant gene expression changes between the WT CM and BKO CM. Some of the genes affected are probably direct targets of TOP2B whilst others may reflect gene expression changes due to the altered regulation of genes targeted by TOP2B.

Importantly, we showed that gene expression changes after a short exposure of WT CM cells to topobexin, a new selective TOP2B catalytic inhibitor partially phenocopies the BKO genotype. The availability of a small molecule inhibitor with selectivity for TOP2B opens the possibility of using WT CM plus or minus topobexin to determine the mechanism by which reduction of TOP2B activity mediates cardio protection following anthracycline exposure. Topobexin has been shown to protect against cardiac damage caused by anthracyclines (Kubeš et al., 2025) paving the way for development of TOP2B specific drugs for cardio protection. In addition, the availability of a small molecule selective TOP2B catalytic inhibitor that phenocopies a knockout may facilitate studies of the role of TOP2B in other cell lines without the need to produce a gene knockout, but further work would be needed to confirm this. Caveats of this study are that only one clonal line was sequenced. That the drug exposures are short term inhibitions of TOP2 whereas the knockout is a long term. And it is possible the inhibitors may trigger transcriptional stress responses, chromatin effects or potential off target activities that would not necessarily be recapitulated in the chronic knockout setting.

In conclusion, this report analyzes the role of TOP2B in hiPSC-derived CM using a combination of CRISPR-Cas9 knockout and pharmacological inhibition (topobexin and dexrazoxane), with RNA-seq-based transcriptomic profiling. The central findings are that (i) TOP2B is dispensable for CM differentiation, (ii) TOP2B loss leads to reproducible transcriptional changes in CM, and (iii) the selective TOP2B inhibitor topobexin partially phenocopies genetic deletion of TOP2B. One limitation of this study is the four independent replicates on which the RNA-seq was carried were all derived from one iPSC clone, 18D. A second limitation is we used the appearance of sheets of beating cells as our measure of CM differentiation, we did not analyse calcium transient recordings and ventricular-like action potential assessment using FluoVolt. We found that hiPSC-derived CM lacking TOP2B, or treated with a selective catalytic inhibitor, exhibit characteristic cardiac gene expression, while also revealing distinct transcriptional alterations associated with TOP2B loss or inhibition. This system enables the study of TOP2B function in a human cardiac context and offers a flexible platform for investigating how its modulation impacts CM biology. This may be used in future studies on cardiac development, epigenetic regulation, and cardiotoxicity mechanisms, including those induced by chemotherapeutic agents. However, such studies were beyond the scope of this report.

## MATERIALS AND METHODS

### Cell culture

hiPSCs were derived from unaffected fibroblasts purchased from Lonza a commercial supplier. Undifferentiated hiPSC cells were maintained in Geltrex (Thermo Fisher)-coated wells of six-well plates in mTeSR1 medium (StemCell Technologies). For the first 24 h after plating the medium contained 10 μg/ml ROCK inhibitor (Y27632, StemCell Technologies), after which the medium was replaced with mTeSR1 without ROCKi.

### CRISPR-Cas9 deletion of TOP2B

TOP2B was deleted from hiPSC using CRISPR-Cas9. The gRNA targeting exon 1 (see Fig. S1A) was cloned into plasmids as described in Khazeem et al. (2022). For transfection, 2×10^6^ cells were centrifuged (115× ***g***, 3 min), resuspended in electroporation solution (Amaxa Human Stem Cell Nucleofector Kit, Lonza), and transferred to a cuvette. Plasmids were added, and nucleofection was performed using the Amaxa Nucleofector™ II, program A-23. Cells were then plated on a Geltrex-coated six-well plate, and conditioned mTeSR1 (StemCell Technologies) medium (1:1 fresh and pre-used, filtered through a 0.22 μm PES filter) was added. After 48 h, cells were detached with Accutase (Biosera) and mixed with 2 ml FBS. Clumps were removed using a 40 μm sieve, and single-cell suspensions were transferred to flow cytometry tubes. Hoechst 33342 (Thermo Fisher Scientific, 0.1 μl/ml) was added for nuclear staining. Cells were sorted based on GFP expression into Geltrex-coated 96-well plates containing 100 μl conditioned mTeSR1 medium with ROCK inhibitor (10 μg/ml). After 24 h, the medium was replaced with fresh mTeSR1 and changed every other day. Visible colonies appeared after about 1 week, and by 4 weeks, genotyping could be performed. Screening for TOP2B knockout clones was performed using PCR genotyping and confirmed by DNA sequencing and western blotting (see Fig. S1B,C).

### Immunodetection of TOP2B

Pellets from 1×10^6^ hiPSC were resuspended in extraction buffer (10 mM MgCl_2_, 50 mM Tris-HCl pH 7.4, 4 mM DTT) with 0.05% protease inhibitors (Proteoloc Expedeon, Abcam), then treated with 0.25% SDS and 50 μg/ml DNase I (Thermo Fisher Scientific). After incubation on ice (10–30 min), an equal volume of solubilization buffer (2% SDS, 20% glycerol, 5% β-mercaptoethanol, 0.6 M Tris-HCl pH 7.2) was added, and samples were heated at 68°C for 10 min. Each sample was mixed with 20 μl of sample buffer, heated at 68°C for 5–10 min, and loaded onto ten-well gradient SDS-PAGE gels. Electrophoresis was run in Tris-HEPES-SDS buffer (NuSep) at 120 V for 80 min. Proteins were transferred to nitrocellulose membranes using wet transfer (25 mM Tris, 192 mM glycine, 20% methanol) on ice at 110 V and 500 mA for 70 min. Membranes were blocked overnight at 4°C in 5% milk in 0.1% TBS-T. The next day, they were incubated with primary anti-TOP2B antibody (MAB6348, 1:500, R&D Systems) and anti-β-actin antibody (NB600-501, 1:5000, Novusbio) for 1 h at room temperature, then washed with 0.1% TBS-T (2×1 min, 1×10 min, repeated twice). After incubation with HRP-conjugated secondary antibody (NXA931, 1:5000, GE Healthcare) for 1 h at room temperature, membranes were washed again using the same protocol. Signal was detected using Clarity ECL substrate (Bio-Rad) and imaged with a C-DiGit blot scanner (Li-Cor) (Fig. S1C).

### Cardiac differentiation of hiPSC

Differentiation was initiated at 70% confluence derived from (Lian et al., 2013) by replacing mTeSR1 (StemCell Technologies) medium with RPMI 1640 (Gibco) supplemented with B27 without insulin (Thermo Fisher Scientific), containing growth factors 10 μM CHIR 99021 (Tocris Bioscience) and 10 ng/ml Activin A (Tocris Bioscience). Cells were then treated following the protocol outlined in Table 1. Spontaneous contractility appeared after 8 days or later.

### Sample preparation for differential gene expression analysis

Cells were cultured as in the previous CM differentiations, using six-well plates with independent differentiations in each well. Total RNA was prepared using an RNase easy mini kit (Qiagen). Samples were prepared from undifferentiated or differentiated cells (beating sheets of CM cells). Additionally, total RNA samples were prepared from differentiated CM cells after treatment with ICRF-187 (100 μM for 3 h immediately prior to cell lysis) or treatment with topobexin (10 μM for 3 h immediately prior to cell lysis). The treatment times and concentrations were based on previous studies. Four replicates were prepared for each condition. For each replicate RNase easy prep was prepared from a single well of a six-well plate. To check the RNA it was run on an Agilent TapeStation to determine the concentration and assess sample integrity using the RIN number.

RNA libraries for RNA-seq were prepared using Illumina Stranded mRNA Prep, Ligation library preparation protocol. Single-end sequencing was performed using an Illumina NovaSeq 6000 sequencer. 30 million reads were done for each sample. Transcript-level quantification was performed using Salmon (Patro et al., 2017). Subsequent analysis steps employed R software (version 4.3). PCA analysis employed DESeq2 (Love et al., 2014). The GEO accession number for RNA-seq data reported here is GSE262148.

### Ethics statement

Human iPSCs were derived from unaffected fibroblasts purchased from Lonza a commercial supplier. Ethical permission for the use of the iPSC line was not required by our institution since Lonza have an established ethical governance framework.

## Supplementary Material

10.1242/biolopen.062592_sup1 Supplementary information

Table S1.

Table S2.
