# Supplementary material for: Analysis of gene expression changes upon topobexin treatment and TOP2B-knockout in hiPSC-derived cardiomyocytes
Source: Biol Open. 2026 Aug 12;15(8):bio062592. doi: 10.1242/bio.062592 (PMC13552481; doi:10.1242/bio.062592)
Supplement: Supplementary file 1 [file biolopen-15-062592-s1.pdf]

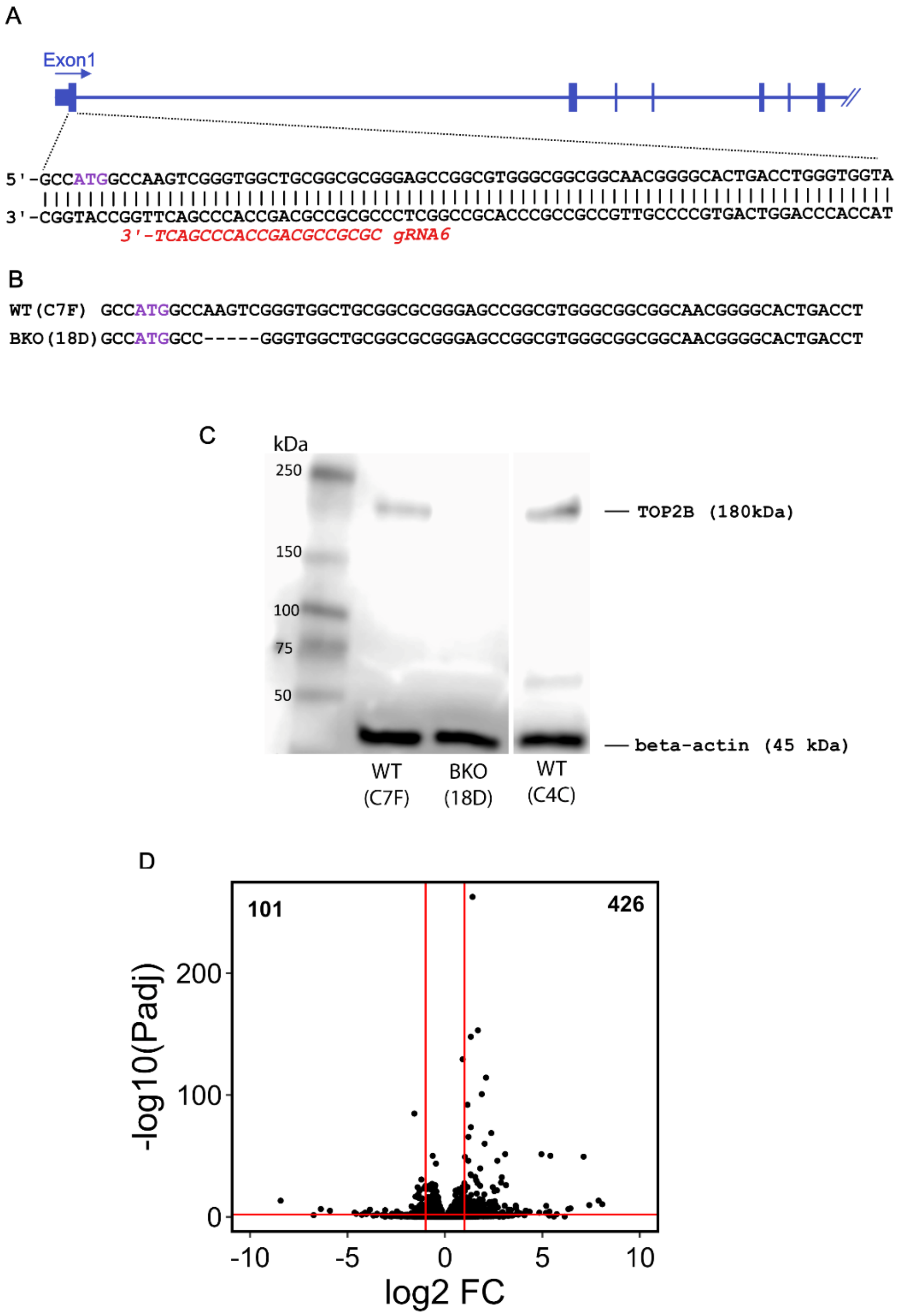

Fig. S1. CRISPR/Cas9 silencing of TOP2B in human iPSC cells. (A) (Upper) 5'-region of the TOP2 locus illustrating the first seven exons. (Lower) DNA sequence on the 5'-coding region of exon 1 with silencing gRNA sequence shown in red. (B) DNA sequences of WT (clone CF7) and mutated (TOP2B null, BKO) clone 18D used in this study. **(C)** Western blot confirming the absence of TOP2B expression in clone 18D. **(D)** Volcano plot illustrating the transcriptional changes associated with TOP2B gene inactivation in iPSC cells. Genes upregulated in 18D (BKO, TOP2B null) cells are to the right and downregulated genes to the left. The cut-offs (red lines) are *P*adj < 0.05, Log2 Fold change in gene expression >1 or <-1.

Biology Open • Supplementary information

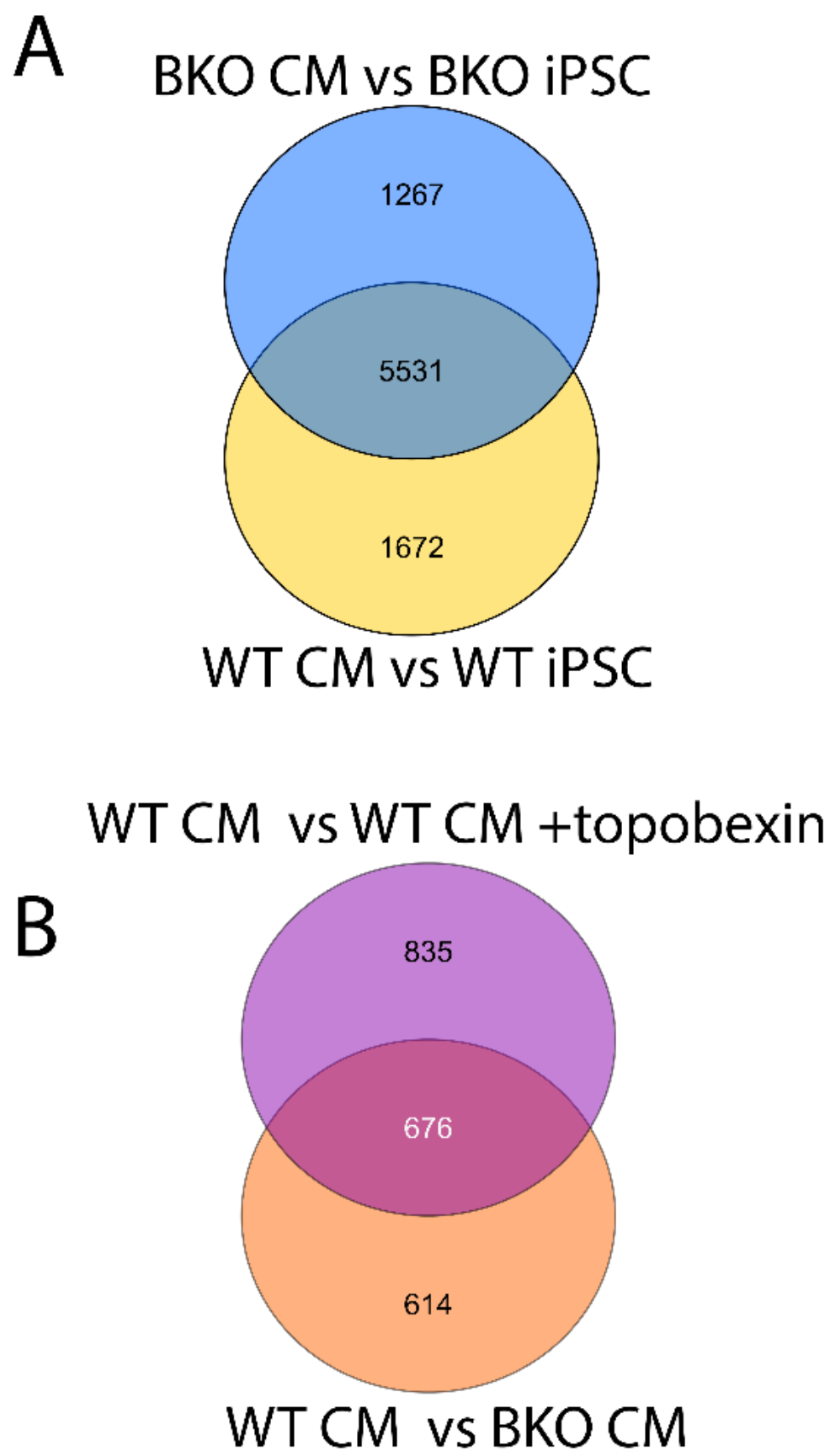

Fig. S2. (A) Venn diagram comparing genes that change in expression in WT and TOP2B null cells (BKO) during differentiation to CMs. **(B)** Venn diagram comparing differences in gene expression in WT vs TOP2B null CM cells and in WT CM cells vs WT CM cells treated with topobexin. Cut-offs are gene expression change of 2 fold or greater (in either direction) and *Padj*<0.05.

Table S1.

Available for download at
https://journals.biologists.com/bio/article-lookup/doi/10.1242/bio.062592#supplementary-data

Table S2.

Available for download at
https://journals.biologists.com/bio/article-lookup/doi/10.1242/bio.062592#supplementary-data

Biology Open • Supplementary information

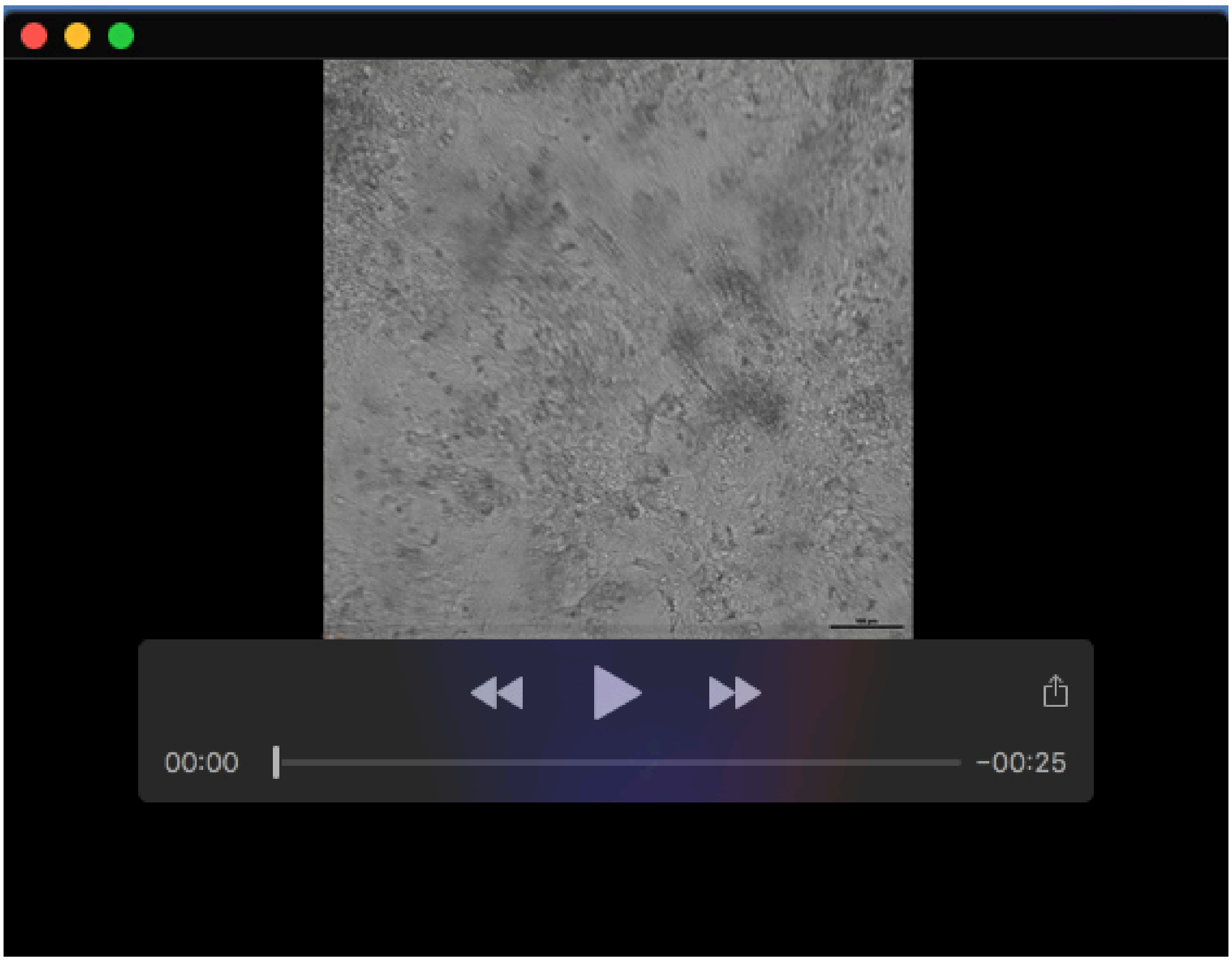

Movie 1.

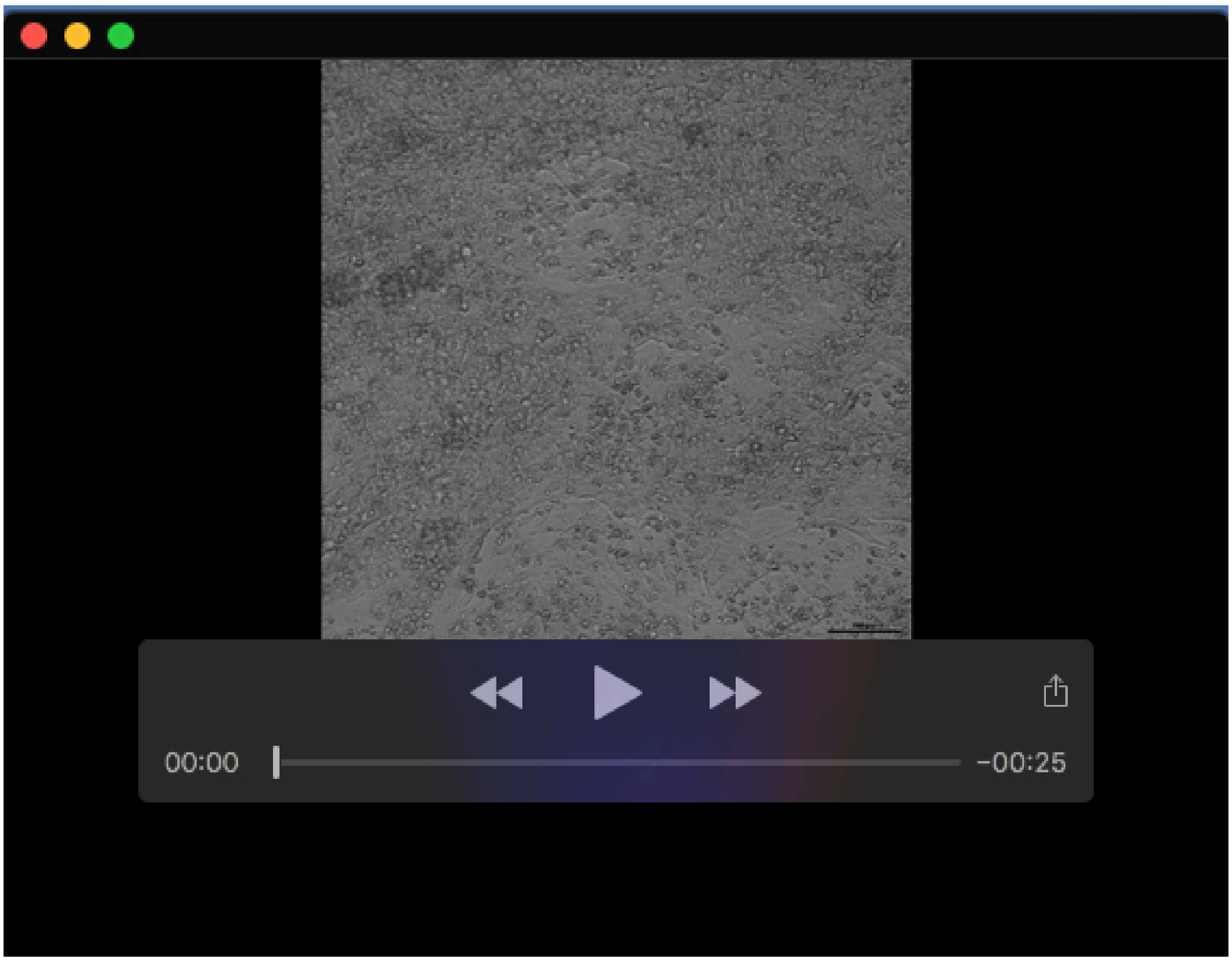

Movie 2.
